# Operationally Defined Sarcopenia Phenotype Among Older Egyptian Outpatients: A Descriptive Cross-Sectional Study

**DOI:** 10.3390/nursrep16090326

**Published:** 2026-09-07

**Authors:** Fatma Magdi Ibrahim

**Affiliations:** 1 Faculty of Nursing, Mansoura University, Mansoura 35516, Egypt; fatma_magdi@mans.edu.eg; 2 RAK College of Nursing, RAK Medical and Health Sciences University, Ras Al Khaimah P.O. Box 11172, United Arab Emirates

**Keywords:** sarcopenia, older adults, functional independence, nutrition, gerontological nursing, nursing assessment, Egypt

## Abstract

**Background/Objectives**: Sarcopenia can compromise mobility and self-care. This study estimated the prevalence of an operationally defined sarcopenia phenotype among older Egyptian outpatients and described characteristics relevant to nursing assessment; Orem’s Self-Care Deficit Nursing Theory framed interpretation but was not tested. **Methods**: This cross-sectional study consecutively recruited 320 adults aged ≥60 years from outpatient clinics at four randomly selected public hospitals in El-Beheira Governorate (August 2024–January 2025). The operational definition, based on the EWGSOP 2010 framework, combined calf circumference <31 cm as a pragmatic muscle-mass proxy with low handgrip strength and/or gait speed ≤ 0.8 m/s. Nutrition and functional independence were assessed using the BMI-based MNA-SF and Lawton-Brody IADL scale. Primary analyses comprised descriptive summaries and Wilson 95% confidence intervals; exploratory Firth-penalized logistic regression models adjusted for age and sex were sensitivity analyses. **Results**: The phenotype was present in 109/320 participants (34.1%; 95% CI, 29.1–39.4%). Prevalence was 2.7% at ages 60–74 years, 16.8% at 75–84 years, and 91.6% at ≥85 years. Nutritional vulnerability, IADL dependence, food insecurity, polypharmacy, and recent hospitalization were descriptively more common among participants meeting the definition. Adjusted models retained several associations but yielded an unstable reversed nutritional estimate and wide confidence intervals. **Conclusions**: The operational phenotype identified a clinically vulnerable outpatient subgroup relevant to nursing and interdisciplinary assessment. The proxy-based definition, clinic sampling, extreme age imbalance, and cross-sectional design preclude confirmation of sarcopenia and independent-association, prognostic, or causal inference.

## 1. Introduction

Sarcopenia is a progressive skeletal muscle disorder characterized by reduced muscle strength, low muscle quantity or quality, and impaired physical performance. It is associated with falls, disability, hospitalization, and mortality in older adults [1,2]. Because sarcopenia affects mobility, endurance, and the physical capacity required for daily activities, it is also directly relevant to nursing assessment and care planning [3].

Reported prevalence varies widely because estimates depend on diagnostic criteria, care setting, case mix, and the method used to assess muscle mass. Systematic reviews and cross-national studies indicate a greater burden in healthcare-seeking and functionally impaired samples than in healthier community samples [4,5,6]. Evidence from Egyptian outpatient settings remains limited.

Two European Working Group on Sarcopenia in Older People (EWGSOP) consensuses are particularly influential. The original 2010 algorithm defined sarcopenia as low muscle mass together with either low muscle strength or low physical performance [7]. EWGSOP2 subsequently placed low muscle strength first and requires low muscle quantity or quality to confirm the diagnosis [2]. The present study was operationalized using the prespecified 2010 algorithm because its data-collection forms and thresholds were established before fieldwork. Because calf circumference was used as a proxy for muscle mass, the resulting estimate is described as operationally defined sarcopenia and should not be interpreted as an EWGSOP2-confirmed diagnosis.

Orem’s Self-Care Deficit Nursing Theory (SCDNT) was applied as an interpretive framework [8]. It did not determine eligibility, the operational sarcopenia definition, or the selected measurement instruments. Instead, it was used to organize the nursing interpretation around therapeutic self-care demands, functional capacity, and basic conditioning factors such as age, nutrition, education, living arrangement, and social resources. IADL was treated as a self-care-relevant functional indicator, not as a direct measure of Orem’s self-care agency. Accordingly, SCDNT was neither operationalized nor formally tested in this cross-sectional study.

The study aimed to estimate the prevalence of an operationally defined sarcopenia phenotype among community-dwelling older adults attending outpatient clinics in Egypt and to describe, without testing independent associations, the demographic, social, nutritional, and functional distributions that may inform nursing assessment.

## 2. Materials and Methods

### 2.1. Design and Reporting

A cross-sectional study was conducted and reported with reference to the Strengthening the Reporting of Observational Studies in Epidemiology (STROBE) statement [9].

### 2.2. Setting and Sampling

At the time of sampling, the eligible sampling frame comprised eight central (secondary-level) public hospitals in El-Beheira Governorate. Four hospitals were selected by simple random sampling from the eight-hospital sampling frame. The selected hospitals served mixed urban and rural catchment areas and provided outpatient services frequently used by older adults, including internal medicine, orthopedic, and rehabilitation clinics.

Within each selected hospital, consecutive recruitment was conducted from August 2024 through January 2025 on Sunday, Monday, Wednesday, and Thursday, corresponding to the scheduled opening days of the participating outpatient clinics. All eligible attendees present on those days were invited until the target sample was reached. Recruitment by hospital was 50, 102, 60, and 108 participants, respectively (total *N* = 320). Specialty-specific recruitment totals were not retained. The sample comprised community-dwelling, hospital-attending older adults; community-dwelling means residing in a private home rather than an institution and does not imply household-based sampling.

### 2.3. Participants

Eligible participants were Egyptian adults aged 60 years or older who lived in private homes, were receiving care in the participating outpatient clinics, could communicate with the research team, and could provide written informed consent. A previous clinical diagnosis of sarcopenia was not required, and recruitment was not restricted by sarcopenia status. Exclusion criteria were major neurologic disorders, implanted medical devices, and dependence on enteral or intravenous feeding.

### 2.4. Variables and Measurements

#### 2.4.1. Operational Sarcopenia Definition

The primary outcome was classified using the prespecified EWGSOP 2010 logic: a low muscle-mass proxy plus either low muscle strength or low physical performance [7]. Low handgrip strength was defined as <30 kg for men and <20 kg for women. Grip strength was measured in the dominant hand using a hand dynamometer; three trials were performed, and the highest value was retained [10]. The archived protocol and field records did not retain the dynamometer manufacturer and model, participant positioning, intertrial rest interval, or calibration procedure and schedule. These omissions limit exact replication and evaluation of measurement reliability and are acknowledged in the limitations.

Low physical performance was defined as usual gait speed ≤ 0.8 m/s over 4 m from a standing start. Two trials were recorded, and the faster performance was retained [2,11]. The archived materials did not preserve details of the walkway markings, timer specification, intertrial rest interval, or rules for assistive-device use. Calf circumference was recorded in centimeters, with <31 cm serving as the prespecified pragmatic proxy for low muscle mass because analyzable bioelectrical impedance analysis or dual-energy X-ray absorptiometry data were unavailable; calf circumference was available for all 320 participants. The tape type, exact anatomical site, participant posture, side measured, number of replicate measurements, allowable difference, and aggregation rule were not documented in retrievable records. These gaps limit exact replication and evaluation of measurement error. EWGSOP2 permits calf circumference as a proxy when instrument-based muscle-mass measurement is unavailable, and <31 cm has been associated with poorer physical function [2,12]. However, this threshold was evaluated primarily in older women, is not sex-specific, and can be influenced by body size, adiposity, edema, and disease. Alternative sex-specific screening cutoffs, such as <34 cm in men and <33 cm in women proposed by AWGS 2019, were developed for Asian populations and are not validated diagnostic thresholds for Egyptian adults [13]. They were not prespecified and were not substituted post hoc for the primary definition; no alternative-threshold sensitivity analysis was performed. Consequently, the outcome cannot confirm low muscle quantity or quality and is consistently termed an operationally defined sarcopenia phenotype rather than confirmed sarcopenia.

#### 2.4.2. Nutritional and Functional Status

Nutritional status was assessed with the Arabic Mini Nutritional Assessment-Short Form (MNA-SF; score range 0–14). The instrument includes five clinical questions and one anthropometric item. Height and weight were available for all participants and were used to calculate BMI; accordingly, the BMI-based anthropometric item was used for every MNA-SF score, and the alternative calf-circumference item was not used [14,15]. Thus, calf circumference did not contribute to both the nutritional score and the operational sarcopenia classification. Scores of 12–14 indicate normal nutritional status, 8–11 indicate risk of malnutrition, and 0–7 indicate malnutrition. Functional status was assessed with the Lawton-Brody IADL scale (range 0–8), with higher scores indicating greater independence [16]. For descriptive presentation, 7–8, 3–6, and 0–2 independent items were labeled >75%, 26–75%, and ≤25% independent, respectively; these were study-defined descriptive groupings rather than validated clinical cutoffs.

#### 2.4.3. Other Variables

Sociodemographic and clinical variables included age, sex, marital status, education, residence, living arrangement, chronic disease, number of medications used per day, hospitalization and surgery during the previous year, and falls. Food insecurity was represented by three separate yes/no items: insufficient money for food, skipped meals, and choosing between food and household bills. These were study-specific indicators rather than a validated composite scale, and no summary score was calculated.

### 2.5. Data Collection and Quality Assurance

The available fieldwork record indicates that assessors received pre-fieldwork demonstration and supervised practice for handgrip strength, gait speed, and circumference measurement. However, the number and professional background of assessors, training duration, quantitative repeatability or inter-rater reliability data, periodic quality-control schedule, and protocol for resolving discrepant measurements were not preserved. No inter-rater reliability coefficient can therefore be reported.

### 2.6. Sample Size

The recruitment target of 320 participants was prespecified for estimating a single population proportion. However, the archived protocol did not retain the expected prevalence, absolute precision, unadjusted minimum, or allowance for nonresponse or incomplete data used to derive that target. These assumptions were not reconstructed post hoc. With 109 cases among 320 participants, the observed estimate was 34.1% (Wilson 95% CI, 29.1–39.4%); this interval describes achieved precision and is not a substitute for the missing a priori calculation record.

### 2.7. Statistical Analysis

Descriptive summaries were generated using IBM SPSS Statistics for Windows, version 21.0 (IBM Corp., Armonk, NY, USA). Continuous variables were summarized as mean ± standard deviation or median [interquartile range], as appropriate; categorical variables were summarized as number (percentage). Overall and subgroup prevalence estimates were reported with Wilson 95% confidence intervals. Because age produced sparse and near-separated outcome cells, exploratory sensitivity analyses used Firth-penalized logistic regression, which yields finite estimates under separation [17,18]. Separate parsimonious models assessed each clinically relevant characteristic while adjusting for age (continuous, per 10 years) and sex. Adjusted odds ratios (aORs), 95% confidence intervals derived from the penalized information matrix, and two-sided Wald p values were reported. Defining components of the operational phenotype were excluded as predictors. These models were used to evaluate the robustness of descriptive contrasts and were not interpreted as predictive or causal analyses.

### 2.8. Ethical Considerations

The protocol was approved by the Damanhur Nursing Research Committee (approval no. 57-b). Written informed consent was obtained from every participant before data collection.

## 3. Results

### 3.1. Participant Flow and Sample Characteristics

After data cleaning, 320 unique participants with complete primary-outcome data were included. Data were complete for the variables reported in Table 1, Table 2, Table 3 and Table 4. Screening logs for eligible nonparticipants were not retained; therefore, the numbers approached and declining participation and the response rate could not be calculated. This limits assessment of nonresponse and selection bias.

Participants had a mean age of 78.4 ± 10.1 years; 59.4% were men, 65.6% were married, 50.0% were unable to read or write, and 85.6% lived with family. Participants meeting the operational definition were, on average, 15.8 years older than those who did not meet it. Women, widowed participants, those unable to read or write, participants using ≥5 medications/day, those hospitalized in the previous year, and those reporting food insecurity comprised larger proportions of the operational-phenotype group than of the comparison group (Table 1). These are unadjusted descriptive distributions, not evidence of independent association, prediction, or risk.

### 3.2. Anthropometric, Functional, and Nutritional Characteristics

Participants meeting the operational sarcopenia definition had lower descriptive mean values for height, weight, mid-arm circumference, calf circumference, gait speed, and handgrip strength, whereas mean BMI values were similar (Table 2). In the complete sample, 21.2% had low handgrip strength, 37.5% had calf circumference <31 cm, and 32.2% had low physical performance. Calf circumference, grip strength, and gait speed are components of the operational definition; their group distributions are structurally constrained and are presented only to document classification, without *p* values or claims of association.

The overall median IADL independence count was 3 [2–4]. It was 0 [0–2] among participants meeting the operational definition and 3 [3–6] among other participants. The overall median MNA-SF score was 8 [5–9], with group-specific medians of 5 [4–8] and 9 [7–10], respectively (Table 2). These distributions are descriptive and strongly confounded by age.

Classification audit: the 120 participants with calf circumference <31 cm were separated into four mutually exclusive patterns. The union of low grip and low physical performance among these participants yielded the 109 operational cases; the remaining 11 had low calf circumference alone and did not meet the definition.

### 3.3. Prevalence Across Selected Subgroups

The overall prevalence estimate was 34.1% (109/320; 95% CI 29.1–39.4). Descriptive prevalence was 2.7% among participants aged 60–74 years, 16.8% among those aged 75–84 years, and 91.6% among those aged ≥85 years. This unusually large gradient is not a population age effect and may reflect clinic-based case mix, the calf-circumference proxy, and overlap of age with nutritional, functional, and comorbidity profiles. Prevalence estimates for sex, marital status, education, living arrangement, and MNA-SF class are provided only as subgroup descriptions (Table 4).

### 3.4. Age- and Sex-Adjusted Sensitivity Analyses

Firth-penalized logistic regression materially changed several unadjusted patterns, indicating substantial age confounding (Table 5). After adjustment for age and sex, the food-insecurity indicators, inability to read or write, widowhood, and lower IADL independence retained associations with the operational phenotype. Polypharmacy, recent hospitalization, and living alone were not independently associated. The adjusted direction for MNA-SF malnutrition was reversed, indicating instability arising from the strong age structure and correlated vulnerabilities; it should not be interpreted causally.

## 4. Discussion

### 4.1. Principal Findings

Approximately one-third of this hospital-attending sample met the operational sarcopenia phenotype definition, with an exceptionally steep descriptive age gradient. Participants meeting the definition also had less favorable nutritional, functional, social, and clinical distributions. These observations identify a subgroup with multiple nursing and interdisciplinary care needs but do not establish whether any characteristic preceded, resulted from, or was independently associated with the classification.

### 4.2. Comparison with Existing Evidence

The 34.1% estimate exceeds many pooled estimates from general community samples but is plausible for an older, healthcare-seeking population. International syntheses show marked heterogeneity by setting and case definition [4,5,6], and clinic- or day-hospital samples tend to have a greater burden than healthier population-based cohorts [19]. Direct numerical comparison nevertheless requires caution because the present study used calf circumference rather than an instrument-based measure of muscle mass.

Age showed the clearest descriptive gradient. Although age-related losses of muscle reserve and neuromuscular performance are biologically plausible [2,20], prevalence rising from 2.7% at ages 60–74 years to 91.6% at ages ≥85 years requires cautious methodological interpretation. Only 3 participants in the youngest stratum met the operational definition, whereas only 8 in the oldest stratum did not. Clinic-based recruitment, the calf-circumference proxy, and the overlap of age with functional limitation, nutritional vulnerability, multimorbidity, inflammation, and medication burden may magnify the gradient. Consequently, the subgroup estimates should not be generalized as age-specific population prevalence or interpreted as an independent age effect.

Nutritional vulnerability was descriptively concentrated among participants meeting the operational definition. The pattern is compatible with the recognized interrelationship among inadequate intake, loss of muscle mass and strength, and reduced physical performance [21,22]. The BMI-based MNA-SF was used, so calf circumference did not directly overlap with the nutritional score. Nevertheless, age and disease burden could account for much of the observed distribution; no independent nutrition-sarcopenia association is claimed.

IADL dependence was substantial among participants meeting the operational definition. This is important for nursing because IADL limitations can affect medication management, food preparation, finances, transportation, and the ability to follow therapeutic recommendations. The finding aligns with reports linking sarcopenia to disability and loss of independence [20], while avoiding the stronger claim that Orem’s self-care agency was directly measured.

Widowhood, lower educational attainment, and food-insecurity indicators were descriptively more frequent among participants meeting the operational definition. Because these distributions were not adjusted for age or sex, they are not interpreted as associated factors, predictors, or risk factors. They may still identify practical domains for individualized nursing assessment, family engagement, nutrition support, and referral to social resources.

The separation-robust sensitivity analyses indicated that age was a major confounder. Food-insecurity indicators, lower IADL independence, inability to read or write, and widowhood retained adjusted associations, whereas polypharmacy, recent hospitalization, and living alone did not. The reversed adjusted nutritional estimate and wide confidence intervals for some variables demonstrate model instability and correlated vulnerability domains; therefore, these results are interpreted as sensitivity findings rather than causal or predictive effects.

### 4.3. Orem-Informed Nursing Implications

From an Orem-informed perspective, reduced strength and mobility may increase the gap between therapeutic self-care demands and the individual’s ability to meet them. The present study did not evaluate a case-finding program, intervention feasibility, or intervention effectiveness. Its observations support consideration of an integrated nursing review when low grip strength, slow gait, or other concerns are detected. Such review may include nutrition, medication burden, IADL performance, food access, comorbidity, and available family support. Any subsequent plan should be individualized to the patient’s clinical condition and delivered through interdisciplinary collaboration with medical, dietetic, pharmacy, physiotherapy, rehabilitation, and social-care professionals as appropriate. Evidence linking polypharmacy with sarcopenia provides additional rationale for considering medication review within this pathway [23].

These implications represent a reasoned application of the observations rather than an empirically tested nursing model. Future studies should directly measure self-care agency and prospectively evaluate the feasibility, effectiveness, acceptability, and safety of Orem-guided supportive-educative interventions.

### 4.4. Strengths and Limitations

Strengths include recruitment from four hospitals, complete primary-outcome data, an explicit audit of the component combinations used for classification, consistent application of prespecified grip, gait, and calf-circumference thresholds, and a clear distinction between nursing-theory interpretation and theory testing.

Several limitations warrant emphasis. First, the descriptive cross-sectional design precludes temporal, causal, predictive, or independent-association inference. Second, outpatient recruitment in one governorate limits generalizability and creates potential referral, clinic-day, and nonresponse bias; the numbers approached and declining participation were unavailable. Third, calf circumference is an accessible but imperfect muscle-mass proxy. The <31 cm threshold is not sex-specific, was evaluated primarily in older women, and may be distorted by body size, adiposity, edema, vascular disease, chronic disease, or inflammation [2,12]. Alternative sex-specific screening thresholds have not been validated for Egyptian adults, and no alternative-cutoff sensitivity analysis was prespecified [13]; the outcome is therefore not equivalent to EWGSOP2-confirmed sarcopenia. Fourth, key procedural metadata for dynamometry, gait timing, calf-circumference measurement, assessor agreement, and the original sample-size assumptions were not retained, limiting reproducibility and preventing full evaluation of measurement error and study-size planning. Fifth, participants were clustered within four hospitals, but site-level and cluster-adjusted estimates were unavailable. Sixth, although Firth models addressed separation and adjusted for age and sex, residual confounding, correlated vulnerabilities, multiple sensitivity models, and wide confidence intervals limit independent interpretation. Seventh, food insecurity was represented by three study-specific yes/no items rather than a validated scale, and detailed comorbidity profiles were unavailable. Finally, IADL performance was used as a self-care-relevant functional indicator rather than a direct measure of Orem’s theoretical constructs.

## 5. Conclusions

In this clinic-based sample, the operationally defined sarcopenia phenotype identified a subgroup with substantial nutritional vulnerability, IADL dependence, medication burden, recent hospitalization, and social disadvantage. These descriptive observations may inform individualized nursing assessment and interdisciplinary referral. However, the calf-circumference proxy, selected outpatient sample, strong age imbalance, and descriptive cross-sectional analysis preclude a confirmed diagnosis and any conclusions about independent associations, prognosis, or causality. Future studies should use instrument-based muscle-mass measurement, direct assessment of self-care agency, detailed comorbidity and inflammatory measures, and prespecified age- and sex-adjusted separation-robust models.

## Figures and Tables

**Table 1:** Sociodemographic and Clinical Characteristics by Operational Sarcopenia Status.

Characteristic	Overall (*N* = 320)	Does Not Meet Definition (*n* = 211)	Meets Definition (*n* = 109)
Age, years	78.4 ± 10.1	73.0 ± 7.0	88.8 ± 6.2
**Age group**			
60–74	112 (35.0)	109 (51.7)	3 (2.8)
75–84	113 (35.3)	94 (44.5)	19 (17.4)
≥85	95 (29.7)	8 (3.8)	87 (79.8)
**Sex**			
Male	190 (59.4)	136 (64.5)	54 (49.5)
Female	130 (40.6)	75 (35.5)	55 (50.5)
**Marital status**			
Married	210 (65.6)	167 (79.1)	43 (39.4)
Widowed	79 (24.7)	26 (12.3)	53 (48.6)
Divorced	21 (6.6)	12 (5.7)	9 (8.3)
Single	10 (3.1)	6 (2.8)	4 (3.7)
**Residence**			
Urban	163 (50.9)	104 (49.3)	59 (54.1)
Rural	157 (49.1)	107 (50.7)	50 (45.9)
**Education level**			
Unable to read or write	160 (50.0)	79 (37.4)	81 (74.3)
Read and write	80 (25.0)	60 (28.4)	20 (18.3)
Primary	22 (6.9)	19 (9.0)	3 (2.8)
Preparatory	6 (1.9)	5 (2.4)	1 (0.9)
Secondary	24 (7.5)	21 (10.0)	3 (2.8)
University	28 (8.8)	27 (12.8)	1 (0.9)
**Living arrangement**			
With family	274 (85.6)	186 (88.2)	88 (80.7)
Alone	46 (14.4)	25 (11.8)	21 (19.3)
**Any chronic disease**			
No	19 (5.9)	15 (7.1)	4 (3.7)
Yes	301 (94.1)	196 (92.9)	105 (96.3)
**Medications/day**			
0	40 (12.5)	30 (14.2)	10 (9.2)
<5	67 (20.9)	53 (25.1)	14 (12.8)
≥5	213 (66.6)	128 (60.7)	85 (78.0)
**Hospitalized in past year**			
No	184 (57.5)	134 (63.5)	50 (45.9)
Yes	136 (42.5)	77 (36.5)	59 (54.1)
**Surgery in past year**			
No	255 (79.7)	175 (82.9)	80 (73.4)
Yes	65 (20.3)	36 (17.1)	29 (26.6)
**Falls in past year**			
No	191 (59.7)	126 (59.7)	65 (59.6)
Yes	129 (40.3)	85 (40.3)	44 (40.4)
**Food insecurity: insufficient money for food**			
No	157 (49.1)	147 (69.7)	10 (9.2)
Yes	163 (50.9)	64 (30.3)	99 (90.8)
**Food insecurity: skipped meals**			
No	164 (51.2)	153 (72.5)	11 (10.1)
Yes	156 (48.8)	58 (27.5)	98 (89.9)
**Food insecurity: chose food versus bills**			
No	176 (55.0)	161 (76.3)	15 (13.8)
Yes	144 (45.0)	50 (23.7)	94 (86.2)

Note. Values are *n* (column %) unless otherwise stated; age is mean ± SD. The table is descriptive. No hypothesis-test *p* values are presented because the marked age imbalance prevents interpreting between-group differences as independent associations.

**Table 2:** Anthropometric, Diagnostic, Functional, and Nutritional Characteristics by Operational Sarcopenia Status.

Measure	Overall (*N* = 320)	Does Not Meet Definition (*n* = 211)	Meets Definition (*n* = 109)
Height, cm	161.7 ± 8.9	163.2 ± 8.8	159.0 ± 8.5
Weight, kg	60.6 ± 16.5	62.7 ± 12.5	56.4 ± 21.8
Body mass index, kg/m^2^	23.0 ± 5.4	23.5 ± 3.9	22.1 ± 7.4
Mid-arm circumference, cm	29.9 ± 7.5	31.4 ± 5.6	26.9 ± 9.5
Calf circumference, cm	34.7 ± 5.3	37.8 ± 3.4	28.5 ± 1.1
Gait speed, m/s	0.96 ± 0.30	1.14 ± 0.16	0.62 ± 0.17
Handgrip strength, kg	33.3 ± 11.2	37.6 ± 7.3	25.1 ± 12.6
**Underweight (BMI < 18.5 kg/m^2^)**			
No	309 (96.6)	211 (100.0)	98 (89.9)
Yes	11 (3.4)	0 (0.0)	11 (10.1)
**Low handgrip strength**			
No	252 (78.8)	211 (100.0)	41 (37.6)
Yes	68 (21.2)	0 (0.0)	68 (62.4)
**Low calf circumference (<31 cm)**			
No	200 (62.5)	200 (94.8)	0 (0.0)
Yes	120 (37.5)	11 (5.2)	109 (100.0)
**Low physical performance**			
No	217 (67.8)	211 (100.0)	6 (5.5)
Yes	103 (32.2)	0 (0.0)	103 (94.5)
IADL independent items (0–8), median [IQR]	3 [2–4]	3 [3–6]	0 [0–2]
**IADL category**			
>75% independent	43 (13.4)	43 (20.4)	0 (0.0)
26–75% independent	169 (52.8)	147 (69.7)	22 (20.2)
≤25% independent	108 (33.8)	21 (10.0)	87 (79.8)
MNA-SF score (0–14), median [IQR]	8 [5–9]	9 [7–10]	5 [4–8]
**MNA-SF class**			
Normal	41 (12.8)	39 (18.5)	2 (1.8)
At risk	125 (39.1)	95 (45.0)	30 (27.5)
Malnourished	154 (48.1)	77 (36.5)	77 (70.6)

Note. Continuous anthropometric and performance variables are mean ± SD. IADL and MNA-SF scores are median [IQR]. Other values are *n* (column %). The table is descriptive; no *p* values are shown. Calf circumference, handgrip strength, and gait speed contribute to the operational definition and are not interpreted as external correlates. BMI, body mass index; IADL, Instrumental Activities of Daily Living; MNA-SF, Mini Nutritional Assessment-Short Form.

**Table 3:** Mutually Exclusive Component Combinations Used to Derive the Operational Phenotype.

Low Calf Circumference	Low Grip	Slow Gait	Operational Classification	*n* (%)
No	No	No	Does not meet definition	200 (62.5)
Yes	No	No	Does not meet definition	11 (3.4)
Yes	Yes	No	Meets definition	6 (1.9)
Yes	No	Yes	Meets definition	41 (12.8)
Yes	Yes	Yes	Meets definition	62 (19.4)
Total				320 (100.0)

Note. Low calf circumference was <31 cm; low grip was <30 kg for men or <20 kg for women; slow gait was ≤0.8 m/s. Percentages use *N* = 320 as the denominator. No participant without low calf circumference met the operational definition.

**Table 4:** Operationally Defined Sarcopenia Phenotype Prevalence across Selected Subgroups.

Group	Cases/Total	Prevalence (%)	95% CI
Overall	109/320	34.1	29.1–39.4
**Age group**			
60–74	3/112	2.7	0.9–7.6
75–84	19/113	16.8	11.0–24.8
≥85	87/95	91.6	84.3–95.7
**Sex**			
Male	54/190	28.4	22.5–35.2
Female	55/130	42.3	34.2–50.9
**Marital status**			
Married	43/210	20.5	15.6–26.4
Widowed	53/79	67.1	56.1–76.4
Divorced	9/21	42.9	24.5–63.5
Single	4/10	40.0	16.8–68.7
**Education level**			
Unable to read or write	81/160	50.6	43.0–58.3
Read and write	20/80	25.0	16.8–35.5
Primary	3/22	13.6	4.7–33.3
Preparatory	1/6	16.7	3.0–56.4
Secondary	3/24	12.5	4.3–31.0
University	1/28	3.6	0.6–17.7
**Living arrangement**			
With family	88/274	32.1	26.9–37.9
Alone	21/46	45.7	32.2–59.8
**MNA-SF class**			
Normal	2/41	4.9	1.3–16.1
At risk	30/125	24.0	17.4–32.2
Malnourished	77/154	50.0	42.2–57.8

Note. Confidence intervals are Wilson 95% intervals. No subgroup hypothesis tests are presented. All estimates are unadjusted, clinic-specific descriptions and do not estimate independent associations. The estimate for participants aged ≥85 years is particularly imprecise as a population indicator because it arises from a selected outpatient sample and a proxy-based definition. MNA-SF, Mini Nutritional Assessment-Short Form.

**Table 5:** Firth Penalized Logistic Regression Sensitivity Analyses Adjusted for Age and Sex.

Characteristic	Contrast	Adjusted OR	95% CI	*p* Value
Malnourished by MNA-SF	vs. normal/at risk	0.07	0.02–0.25	<0.001
IADL independent domains	per 1-domain increase	0.41	0.25–0.69	<0.001
Insufficient money for food	yes vs. no	11.74	4.29–32.14	<0.001
Skipped meals	yes vs. no	10.84	4.00–29.40	<0.001
Chose food instead of bills	yes vs. no	8.64	3.20–23.37	<0.001
Polypharmacy	≥5 medications/day vs. other	0.75	0.29–1.96	0.563
Hospitalized in past year	yes vs. no	0.34	0.07–1.70	0.189
Unable to read or write	vs. any literacy/education	5.82	2.01–16.82	0.001
Widowed	vs. other marital status	11.08	1.94–63.25	0.007
Living alone	vs. with family	1.42	0.21–9.50	0.720

Note. Each row represents a separate Firth model containing age (continuous, per 10 years), sex, and the listed characteristic. OR, odds ratio; CI, confidence interval; IADL, Instrumental Activities of Daily Living; MNA-SF, Mini Nutritional Assessment-Short Form.

## Data Availability

The de-identified data supporting the findings are available from the corresponding author upon reasonable request, subject to institutional and ethical restrictions.

## Public Involvement Statement

No public involvement in any aspect of this research.

## Guidelines and Standards Statement

This manuscript was drafted in accordance with the Strengthening the Reporting of Observational Studies in Epidemiology (STROBE) statement for cross-sectional studies [9].

## References

[1] Coletta G., Phillips S.M. (2023). An elusive consensus definition of sarcopenia impedes research and clinical treatment: A narrative review. Ageing Res. Rev..

[2] Cruz-Jentoft A.J., Bahat G., Bauer J., Boirie Y., Bruyère O., Cederholm T., Cooper C., Landi F., Rolland Y., Sayer A.A. (2019). Sarcopenia: Revised European consensus on definition and diagnosis. Age Ageing.

[3] Polo-Ferrero L., Recio-Rodríguez J.I., González-Manzano S., Sáez-Gutiérrez S., Barbero-Iglesias F.J., Méndez-Sánchez R. (2024). Prevalence and risk factors for sarcopenia in active community-dwelling older adults according to the EWGSOP2 criteria. Geriatr. Nurs..

[4] Petermann-Rocha F., Balntzi V., Gray S.R., Lara J., Ho F.K., Pell J.P., Celis-Morales C. (2022). Global prevalence of sarcopenia and severe sarcopenia: A systematic review and meta-analysis. J. Cachexia Sarcopenia Muscle.

[5] Tan You Mei C., Seah Si Ying S., Lim Yanshan D., Koh S.V., Karthikeyan G., Xia Jiawen O., Low X.L., Quek H.Y., Ong Shuyi A., Low L.L. (2024). Prevalence and factors associated with sarcopenia among older adults in a post-acute hospital in Singapore. PLoS ONE.

[6] Wang L., Chao J., Zhang N., Li X., Li J., Jin S., Tan G., Chen T., Wu Y. (2026). Prevalence and factors associated with sarcopenia in community-dwelling older adults: A systematic review and meta-analysis. Gerontology.

[7] Cruz-Jentoft A.J., Baeyens J.P., Bauer J.M., Boirie Y., Cederholm T., Landi F., Martin F.C., Michel J.P., Rolland Y., Schneider S.M. (2010). Sarcopenia: European consensus on definition and diagnosis. Age Ageing.

[8] Orem D.E. (2001). Nursing: Concepts of Practice.

[9] von Elm E., Altman D.G., Egger M., Pocock S.J., Gøtzsche P.C., Vandenbroucke J.P., STROBE Initiative (2007). The Strengthening the Reporting of Observational Studies in Epidemiology (STROBE) statement: Guidelines for reporting observational studies. PLoS Med..

[10] Roberts H.C., Denison H.J., Martin H.J., Patel H.P., Syddall H., Cooper C., Sayer A.A. (2011). A review of the measurement of grip strength in clinical and epidemiological studies: Towards a standardised approach. Age Ageing.

[11] Studenski S., Perera S., Patel K., Rosano C., Faulkner K., Inzitari M., Brach J., Chandler J., Cawthon P., Barrett-Connor E. (2011). Gait speed and survival in older adults. JAMA.

[12] Rolland Y., Lauwers-Cances V., Cournot M., Nourhashémi F., Reynish W., Rivière D., Vellas B., Grandjean H. (2003). Sarcopenia, calf circumference, and physical function of elderly women: A cross-sectional study. J. Am. Geriatr. Soc..

[13] Chen L.K., Woo J., Assantachai P., Auyeung T.W., Chou M.Y., Iijima K., Jang H.C., Kang L., Kim M., Kim S. (2020). Asian Working Group for Sarcopenia: 2019 Consensus Update on Sarcopenia Diagnosis and Treatment. J. Am. Med. Dir. Assoc..

[14] Abd-Al-Atty M.F., Abou-Hashem R.M., Abd Elaziz K.M. (2012). Prevalence of malnutrition in recently hospitalized elderly in Cairo using a valid and reliable short form of Arabic version of Mini Nutritional Assessment (MNA-SF-A). Middle East J. Age Ageing.

[15] Kaiser M.J., Bauer J.M., Ramsch C., Uter W., Guigoz Y., Cederholm T., Thomas D.R., Anthony P., Charlton K.E., Maggio M. (2009). Validation of the Mini Nutritional Assessment Short-Form (MNA-SF): A practical tool for identification of nutritional status. J. Nutr. Health Aging.

[16] Rasheedy D., Abou-Hashem R.M. (2020). Overestimated functional dependency in older patients: Can we blame gender difference, unneeded assistance, or assessment tools?. Arch. Gerontol. Geriatr..

[17] Firth D. (1993). Bias reduction of maximum likelihood estimates. Biometrika.

[18] Heinze G., Schemper M. (2002). A solution to the problem of separation in logistic regression. Stat. Med..

[19] Connolly K., Cunningham C., Murphy N., Romero-Ortuño R., Horgan F. (2021). Prevalence of sarcopenia and associated factors in older adults attending a day hospital service in Ireland. Eur. Geriatr. Med..

[20] Kitamura A., Seino S., Abe T., Nofuji Y., Yokoyama Y., Amano H., Nishi M., Taniguchi Y., Narita M., Fujiwara Y. (2021). Sarcopenia: Prevalence, associated factors, and the risk of mortality and disability in Japanese older adults. J. Cachexia Sarcopenia Muscle.

[21] Heo S., Yang S.J. (2025). Dietary factors and nutritional guidelines for sarcopenia in older adults: A narrative review. Korean J. Community Nutr..

[22] Zhao R., Dong Y., Zheng Q., Yao J. (2025). Exercise and nutrition strategies for sarcopenia in older adults: Evidence from a network meta-analysis based on EWGSOP and AWGS criteria. Front. Nutr..

[23] Prokopidis K., Giannos P., Reginster J.-Y., Bruyère O., Petrovic M., Cherubini A., Triantafyllidis K.K., Kechagias K.S., Dionyssiotis Y., Cesari M. (2023). Sarcopenia is associated with a greater risk of polypharmacy and number of medications: A systematic review and meta-analysis. J. Cachexia Sarcopenia Muscle.

